# Household Transmission of Enterovirus D68, Washington and Oregon, United States, 2022–2024

**DOI:** 10.3201/eid3207.251733

**Published:** 2026-07

**Authors:** Pavitra Roychoudhury, Erica Wetzler, Anna Elias-Warren, Katherine L. Hoffman, Alex Harteloo, Hyeong Geon Kim, Kevin Kong, Hong Xie, Jolene Gov, Margaret G. Mills, Collrane Frivold, Madison Hollcroft, Mark Drummond, Tara Hatchie, Erica Clark, Brenna Ehmen, Peter D. Han, Luis Gamboa, Sally Grindstaff, Jeremy Stone, Alexander L. Greninger, Lea M. Starita, Christina Lockwood, Janet A. Englund, Marco Carone, Ana A. Weil, Sacha L. Reich, Richard A. Mularski, Mark A. Schmidt, Jennifer L. Kuntz, Allison L. Naleway, Helen Y. Chu

**Affiliations:** Fred Hutchinson Cancer Center, Seattle, Washington, USA (P. Roychoudhury); University of Washington, Seattle (P. Roychoudhury, E. Wetzler, A. Elias-Warren, K.L. Hoffman, A. Harteloo, H.G. Kim, K. Kong, H. Xie, J. Gov, M.G. Mills, C. Frivold, M. Hollcroft, M. Drummond, T. Hatchie, E. Clark, B. Ehmen, P.D. Han, L. Gamboa, S. Grindstaff, J. Stone, A.L. Greninger, L.M. Starita, C. Lockwood, J.A. Englund, M. Carone, A.A. Weil, H.Y. Chu); Brotman Baty Institute for Precision Medicine, Seattle (L. Gamboa); Seattle Children’s Research Institute, Seattle (J.A. Englund); Kaiser Permanente Center for Health Research, Portland, Oregon, USA (S.L. Reich, R.A. Mularski, M.A. Schmidt, J.L. Kuntz, A.L. Naleway)

**Keywords:** Enterovirus D68, viruses, respiratory infections, transmission, genomics, Washington, Oregon, United States

## Abstract

During 2022–2024, a total of 35 of 1,040 households had a distinct symptomatic index case of enterovirus D68; estimated symptomatic secondary infection rate was 13.6%. Sequences from patients within households clustered closely; we observed 0–2 pairwise nucleotide differences between household cases 6–14 days apart.

Enterovirus D68 (EV-D68) typically causes respiratory illness with symptoms such as difficulty breathing, fever, wheezing, and respiratory distress (*1*). Interest in EV-D68 has grown since 2014 because of associations with acute flaccid myelitis in children (*2*) and recent outbreaks in the United States and Europe (*3*). In a descriptive epidemiologic study of the 2014 US outbreak of medically attended EV-D68, children <16 years of age accounted for >80% of severe respiratory illness cases (*4*); however, community-based assessments of EV-D68 incidence and age distribution remain limited. In congregate settings like homeless shelters, EV-D68 transmission occurred primarily in adult and not family shelters (*5*). No specific treatments or vaccines are available for nonpolio enteroviruses, and the epidemiology of EV-D68, including risk factors for household transmission, are not well understood (*6*). 

Few studies have incorporated genomic sequencing to confirm transmission patterns or quantify viral evolution between transmission events (*7*,*8*). Our study aimed to characterize the epidemiology and household transmission of EV-D68 using specimens and data collected as part of a community-based prospective CASCADIA cohort (*9*).

## The Study

The CASCADIA study included active surveillance for respiratory viruses among enrolled households with children and adults in metropolitan Seattle, Washington, USA, and Portland, Oregon, USA during June 2022–March 2024 (Appendix). Participants completed surveys and self-collected nasal swab samples weekly, regardless of symptoms. We subsequently tested swab specimens from participants who reported symptoms <72 hours of collection (Appendix), as well as specimens with a positive or inconclusive result for SARS-CoV-2, influenza, or respiratory syncytial virus (RSV), by using a multiplex assay for 26 targets, including EV-D68. To rule out false-positive results caused by cross-reactivity of the multiplex assay target described previously (*5*), we selected specimens that tested positive for EV-D68 with relative cycle threshold (Crt) <28 for EV-D68–specific reverse transcription PCR. We sequenced samples by hybridization probe capture using a panel that covers ≈80 viral targets (Appendix).

After excluding 179 single-person households, we included 1,040 multiperson households in our analysis (Appendix Figure 1). We detected a distinct EV-D68 index case in 35 (3.4%) households (Table 1, 2). We identified potential secondary transmission, defined as detection of EV-D68 in a symptomatic household contact 1–14 days after a distinct index case, among 7 (20%) households. Those households included 15 household contacts, 14 of whom were tested because they reported symptoms. The 28 households with unlikely secondary transmission included 51 household contacts, 21 of whom were tested because they reported symptoms. Among 66 household contacts of the 35 identified index cases, 9 contacts were infected; we estimated the symptomatic secondary infection rate for EV-D68 as 13.6% (95% CI 6.8%–27.5%). Median detection interval between index and secondary cases was 7 (range 6–14) days. Households with secondary transmission (n = 7) were less likely to have children <5 years of age than households without secondary transmission (n = 28); 28.6% of the households with secondary transmission had young children, compared with 39.3% of those without secondary transmission (p = 0.38 by Fisher exact test). Compared with households with unlikely secondary transmission, index cases in households with secondary transmission were older (median age 11 vs. 9 years) and more frequently reported >2 acute respiratory illness (ARI) symptoms (100% vs. 68%; p = 0.01 by χ^2^ test). Among households with secondary transmission, median age of household contacts was older (42 vs. 26 years of age) than in households with unlikely secondary transmission. Households with secondary transmission had lower household income than those without (42.9% vs. 78.6% had income lower than the median income threshold). A greater percentage of contacts reported underlying conditions in households with secondary transmission than in households with unlikely secondary transmission (67% vs. 45%). We conducted sensitivity analysis including only households with complete enrollment of all members; we observed no difference (Appendix Table 3).

**Table 1:** Characteristics of index cases in study of household transmission of enterovirus D68, Washington and Oregon, United States, 2022–2024*

Characteristics	Potential secondary transmission†	Unlikely secondary transmission‡	Total
No. index cases	7	28	35
Median age, y (range)	11 (3–34)	8.5 (1–48)	9 (1–48)
Age range			
6 mo–1 y	0	2 (7.1)	2 (5.7)
2–4 y	1 (14.3)	8 (28.0)	9 (25.7)
5–12 y	3 (42.9)	8 (28.6)	11 (31.4)
13–50 y	3 (42.9)	10 (35.7)	13 (37.1)
Sex			
F	3 (42.9)	17 (60.7)	20 (57.1)
M	4 (57.1)	11 (39.3)	15 (42.9)
Race or ethnicity			
Asian	0 (0.0)	1 (3.6)	1 (2.9)
White	3 (42.9)	22 (78.6)	25 (71.4)
Multiracial	3 (42.9)	4 (14.3)	7 (20.0)
Other	1 (14.3)	1 (3.6)	2 (5.8)
Hispanic	0 (0.0)	3 (10.7)	3 (8.6)
Child attending school or daycare	5 (71.4)	18 (64.3)	23 (65.7)
Any smoking	0	0	0
Any underlying conditions§	1 (14.3)	8 (28.6)	9 (25.7)
Masking in public at baseline			
Any	7 (100)	23 (82.1)	30 (85.7)
Never	0	5 (17.9)	5 (14.4)
Relative cycle threshold			
Median (range)	18.8 (13.6–21.8)	16.0 (9.6–22.5)	17.3 (9.6–22.5)
Overall median >17.3	2 (28.6)	17 (60.7)	19 (54.3)
Viral co-detections¶			
Any	7 (100)	26 (92.9)	33 (94.3)
SARS-CoV-2	0	1 (3.6)	1 (2.9)
Adenovirus	1 (14.3)	0 (0)	1 (2.9)
RSV	0	1 (3.6)	1 (2.9)
HPIV	1 (14.3)	0	1 (2.9)
ARI symptoms#			
>2 ARI symptoms	7 (100)	19 (67.9)	26 (74.3)
Cough, rhinorrhea, or both	7 (100)	28 (100)	35 (100)
Any care seeking during illness**	0	3 (10.7)	3 (8.6)
Any behavior change to reduce transmission in the air††	3 (42.9)	11 (39.3)	14 (40.0)
Any behavior change to reduce transmission on surfaces‡‡	3 (42.9)	10 (35.7)	13 (37.1)

*Values are no. (%) except as indicated. ARI, acute respiratory illness; EV, enterovirus; HPIV, human parainfluenza virus; RSV, respiratory syncytial virus. †Occurring when EV-D68 is detected in a secondary household member 1–14 days after index case. ‡Unlikely secondary transmission defined as occurring when only 1 EV-D68 case is detected in the household or when >2 EV-D68 cases are in a household but secondary case detected >14 days after index case. §Underlying conditions include asthma, chronic obstructive pulmonary disease (including chronic bronchitis and emphysema), sleep apnea, heart disease, congenital heart disease, heart failure, Down syndrome, hypertension (high blood pressure), diabetes (high blood sugar), liver condition, weak or failing kidneys, cancer or malignancy, arthritis, stroke, deep vein thrombosis or pulmonary embolism, sickle cell disease or thalassemia, weakened immune system, depression, anxiety, thyroid issues, or other health diagnosis. ¶Rhinovirus target was detected in 32 (91.4%) samples but is not shown because of cross-reactivity between that target and EV-D68 and several other enterovirus species (assay ID Vi99990016_po, Thermo Fisher [https://www.thermofisher.com]). #Symptoms reported <7 days of the individual EV-D68 illness episode’s first positive specimen collection, including fever​, cough​, sore throat​, shortness of breath​, myalgia​, rhinorrhea, or a combination of symptoms. All persons included reported >1 symptom. **Self-reported care seeking defined as seeking healthcare from a healthcare provider during illness. ††Includes masking, sleeping separately, covering cough or sneeze before or during illness episode. ‡‡Includes handwashing, cleaning, disinfecting.

**Table 2:** Characteristics of households and household contacts in study of household transmission of enterovirus D68, Washington and Oregon, United States, 2022–2024*

Characteristics	Potential secondary transmission†	Unlikely secondary transmission‡	Total
No. households	7	28	35
Household density§			
2–4 persons	6 (85.7)	24 (85.7)	30 (85.7)
>5 persons	1 (14.3)	4 (14.3)	5 (14.3)
Housing type			
House, condo, or townhouse	7 (100)	27 (96.4)	34 (97.1)
Missing	0	1 (3.6)	1 (2.9)
Household enrollment			
Median no. participants (range)	3 (2–4)	3 (2–4)	3 (2–4)
No children <5 y	5 (71.4)	17 (60.7)	22 (62.9)
Child <5 y, but no childcare	1 (14.3)	2 (7.1)	3 (8.6)
Child <5 y in childcare¶	1 (14.3)	9 (32.1)	10 (28.6)
Income >$100,000	3 (42.9)	22 (78.6)	25 (71.4)
Smoker in household	1 (14.3)	2 (7.1)	3 (8.6)
Study site			
Kaiser Permanente Northwest	4 (57.1)	12 (42.9)	16 (44.4)
University of Washington	3 (42.9)	16 (57.1)	19 (54.3)
No. household contacts	15	51	66
No. swab samples tested from symptomatic participants	14	20	34
Median age, y (range)	42 (2–48)	38 (3–49)	38.5 (2–49)
Age range			
6 mo–1 y	0	0	1 (1.4)
2–4 y	1 (6.7)	1 (2.0)	1 (1.7)
5–12 y	3 (20.0)	17 (33.3)	22 (31.4)
13–50 y	11 (73.3)	33 (64.7)	45 (64.3)
Female sex at birth	9 (60.0)	30 (58.8)	39 (59.1)
Sex			
F	9 (60.0)	29 (56.9)	38 (57.6)
M	6 (40.0)	21 (41.2)	27 (40.9)
Other	0	1 (2.0)	1 (1.5)
Race or ethnicity			
Asian	2 (13.3)	2 (3.9)	4 (6.1)
White	11 (73.3)	44 (86.3)	55 (83.3)
Multiracial	2 (13.3)	5 (9.8)	7 (10.6)
Hispanic	0	4 (7.8)	4 (6.1)
Any smoking	1 (6.7)	2 (3.9)	3 (4.5)
Any underlying conditions#	10 (66.7)	23 (45.1)	33 (50.0)
Masking in public			
Any	14 (93.3)	48 (94.1)	62 (93.9)
Never	1 (6.1)	3 (5.9)	4 (6.1)

*Values are no. (%) except as indicated. ARI, acute respiratory illness; EV, enterovirus; HPIV, human parainfluenza virus; RSV, respiratory syncytial virus. †Occurring when EV-D68 is detected in a secondary household member 1–14 days after index case. ‡Unlikely secondary transmission defined as occurring when only 1 EV-D68 case was detected in the household or when >2 EV-D68 cases were in a household but secondary case detected >14 days after index case. §Household density represents the number of household members regardless of enrollment in the study; thus, numbers might be greater than the number of participants enrolled in the study, if not all household members were enrolled. ¶Daycare or school attendance among >1 child <5 years in the household as reported at enrollment. #Underlying conditions include asthma, chronic obstructive pulmonary disease (including chronic bronchitis and emphysema), sleep apnea, heart disease, congenital heart disease, heart failure, Down syndrome, hypertension (high blood pressure), diabetes (high blood sugar), liver condition, weak or failing kidneys, cancer or malignancy, arthritis, stroke, deep vein thrombosis or pulmonary embolism, sickle cell disease or thalassemia, weakened immune system, depression, anxiety, thyroid issues, or other health diagnosis.

Samples from adults with either a primary or secondary infection (n = 18) tended to have lower minimum Crt values (mean 16.3, SD 4.27) compared with children 6 months to 4 years of age (n = 11; mean Crt 17.5, SD 3.73) and children 5–17 years of age (n = 15; mean Crt 17.1, SD 3.74). Participants who reported >2 ARI symptoms (n = 35) had similar Crt values (mean 16.8, SD 4.12), compared with those reporting a single symptom (n = 9; mean Crt 16.8, SD 3.11). Household contacts with >2 ARI symptoms (n = 9) had lower Crt (mean 16.4, SD 5.17) compared with index cases (n = 26; mean Crt 17.0, SD 3.81) (Appendix Figure 2).

We recovered high-quality EV-D68 whole-genome sequences for >2 persons within a household in a total of 11 households. All sequenced samples in this study (Appendix Table 1) fell within the B3 clade with other GenBank sequences from the United States and Europe; sequences from the same household clustered closely (Figure; Appendix Figure 3). Among those 11 households, 6 had samples collected from different members (co-primary cases) on the same day and the within-household pairwise nucleotide (nt) distance between sequences was 0–1 (median 0.5) nt. In 4 households, samples were collected 6–14 days apart; the within-household pairwise distance was 0–3 (median 1) nt. In 1 household with samples collected 148 days apart, sequences confirmed distinct introductions into the household. Among the 10 households with samples collected 0–14 days apart, sequences from children tended to be more basal in the tree relative to the sequences from adults in the same household; adult sequences contained >1 additional nucleotide substitutions (Appendix Figure 3), suggesting transmission from child to adult. Because we used a hybridization capture–based method for sequencing, enriching for respiratory viruses (Appendix), we identified a coinfecting pathogen in 3 samples: SARS-CoV-2 in a sample from an adult and adenovirus and human parainfluenza virus in 2 samples from children <5 years of age.

**Figure:**
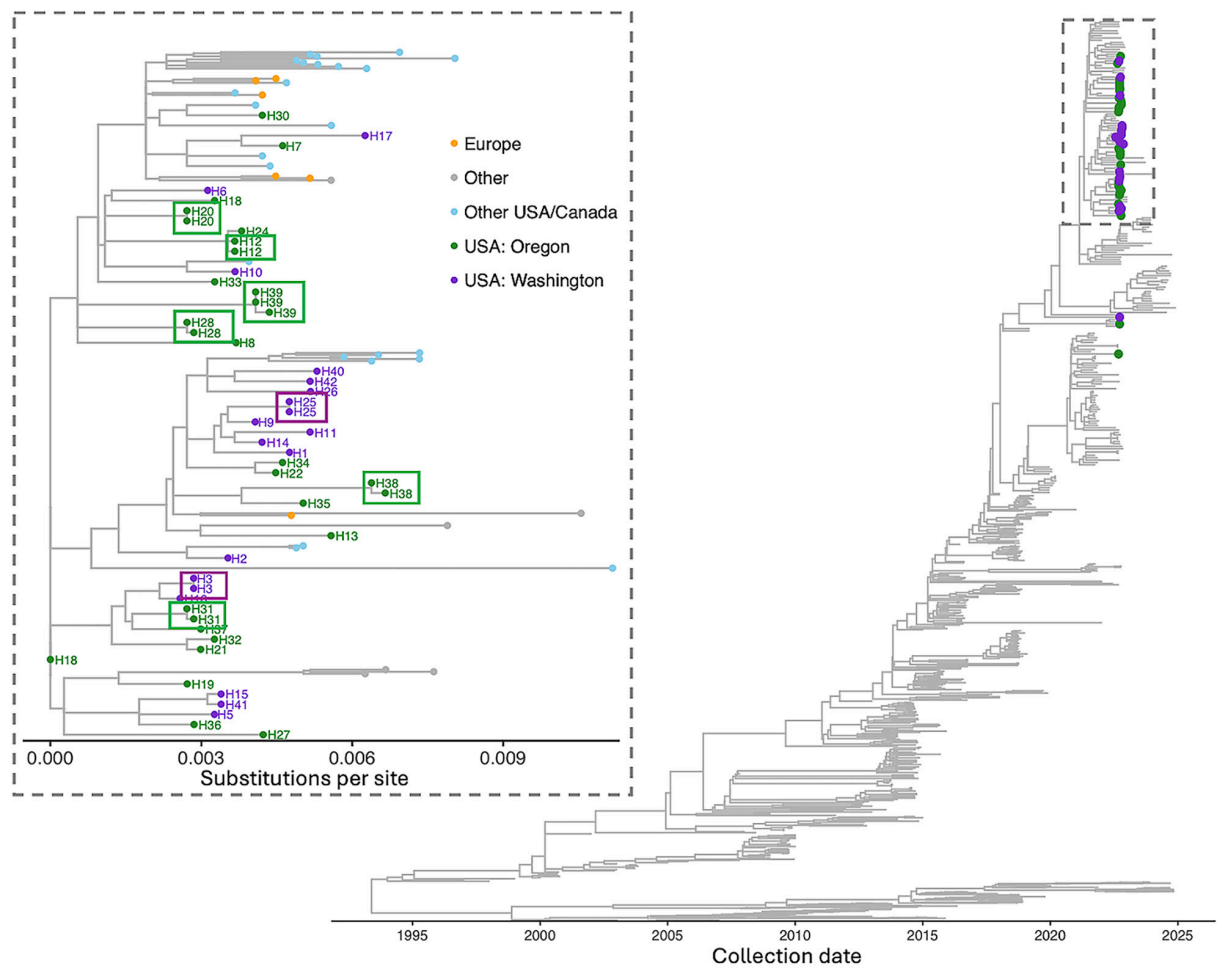
Maximum-likelihood time-resolved phylogenetic tree from study of household transmission of enterovirus D68, Washington (purple tips) and Oregon (green tips), United States, 2022–2024. Tree shows study samples from Washington and Oregon and publicly available global sequences. Inset shows a divergence tree by substitutions per site. Purple and green boxes around tips highlight close clustering of sequences from the same household.

Transmission of symptomatic infection appears to be from younger school-aged children (5–12 years of age) to adults, suggesting introductions to the household from children attending school; however, we are limited by our small sample size. Other limitations of our study were the inability to precisely measure the duration of viral detection or to detect asymptomatic shedding because we collected specimens weekly and tested only swab specimens from symptomatic participants. Because participants swabbed weekly and we did not collect symptom onset dates, we were unable to report serial interval, and we could not determine the exact illness onset date. The estimated median detection interval of 7 days is slightly higher than reported serial intervals for other viruses; a reason could be that our study used weekly sampling, compared with other studies that conducted daily sampling. Our threshold of 14 days for identifying symptomatic secondary infections is supported by our genomic analysis and consistent with other studies that have estimated the duration of EV-D68 shedding in the respiratory tract (*8*); in rare cases, it might miss late transmission events.

## Conclusions

In our 2-year community respiratory virus surveillance study, we found relatively few cases of symptomatic EV-D68 in households compared with other respiratory viruses, such as RSV, in the same cohort of households during an overlapping timeframe, July–November 2022 (*10*) (Appendix Figure 4). Overall, our work adds to the body of literature on enterovirus household transmission and confirms that in households with young children, EV-D68 is a less common source of symptomatic respiratory viral illness than other pathogens such as RSV, influenza virus, and SARS-CoV-2.

This article was preprinted at https://doi.org/10.64898/2026.02.16.26346322.

Appendix Additional information about household transmission of enterovirus D68, Washington and Oregon, United States, 2022–2024.
