# Supplementary material for: Household Transmission of Enterovirus D68, Washington and Oregon, United States, 2022–2024
Source: Emerg Infect Dis. 2026 Jul;32(7):1182–7. doi: 10.3201/eid3207.251733 (PMC13322434; doi:10.3201/eid3207.251733)
Supplement: Appendix — Additional information about household transmission of enterovirus D68, Washington and Oregon, United States, 2022–2024. [file 25-1733-Techapp-s1.pdf]

Article DOI: https://doi.org/10.3201/eid3207.251733

EID cannot ensure accessibility for supplementary materials supplied by authors. Readers who have difficulty accessing supplementary content should contact the authors for assistance.

Household Transmission of Enterovirus D68, Washington and Oregon, United States, 2022–2024

Appendix

Supplemental Methods

Study Design and Population

Study data were from the CASCADIA study, a community-based prospective cohort study among households of infants, children and adults in metropolitan Seattle, Washington and Portland, Oregon, USA (*1*). From 1 June 2022 to March 2024, the study collected data using remote active surveillance of respiratory viruses among enrolled households, with at least weekly at-home collection of nasal swab specimens from participants with and without symptoms. Enrollment was open to people aged ≥6 months to 49 years living in the University of Washington (UW) catchment area (King, Pierce, and Snohomish counties) or the Kaiser Permanente catchment area (northern Oregon and southern Washington). Not all household members were required to enroll in the study. The study protocol was reviewed and approved by the Kaiser Permanente Inter-regional Institutional Review Board, with reliance from University of Washington and Seattle Children's Research Institute (45 C.F.R. part 46.114; 21 C.F.R. part 56.114).

Data Collection

Individuals were eligible for the CASCADIA study if they were between 6 months to 49 years of age during the time of enrollment. After consenting, participants were asked to complete an enrollment survey and an enrollment blood draw. In CASCADIA, a household was defined as individuals living at the same residential address. Not all household members were required to

enroll. Household density (total number of household members regardless of enrollment) was captured at enrollment.

Participants then completed weekly surveys and collected nasal swabs regardless of symptoms, and all swabs were tested for RSV, Influenza, and SARS-CoV-2. Nasal swabs from individuals who reported any new symptom (fever, chills, cough, shortness of breath, fatigue, sore throat, congestion or runny nose, nausea, vomiting, diarrhea, muscle or body aches, headache, and change in smell or taste, persistent pain/pressure in chest, pale/gray/blue lips skin or nail beds, and decreased activity and irritability/crankiness for young non-verbal children) within 72 hours from their weekly swab; as well as swabs that were inconclusive or positive for SARS-CoV-2, Influenza, or RSV also underwent testing on a multiplex PCR panel for 26 pathogens including EV-D68 as described below (*1*,*2*).

PCR Testing and Viral Sequencing

Multiplex PCR testing was performed on selected nasal swab specimens using the OpenArray platform (ThermoFisher) with a custom panel containing 26 targets including EV-D68 as described previously (*1*,*2*). Samples that were positive for the EV-D68 target were selected for this study. RNA was extracted using the MagNAPure 96 DNA and viral nucleic acid small volume kit (Roche Diagnostics), with 200μL input and 50μL elution. Where C_{rt} values are reported, the mean value for each sample was used from two replicates performed on the OpenArray assay.

The EV-D68 target on the OpenArray panel (Vi06439669_s1, ThermoFisher TaqMan Microbe Detection Assays) has documented cross-reactivity to viruses in 16 other taxonomic groups including coxsackie virus, human poliovirus 1 and 2, and other human enteroviruses. Due to this known cross-reactivity of the OpenArray EV-D68 target (*2*), we performed a confirmatory EV-D68-specific real time RT-PCR assay on all samples that had an EV-D68 positive result on OpenArray and no prior sequencing information to confirm EV-D68 positivity. We used the CDC Pan-Enterovirus D68 Real-Time RT-PCR Assay (primer and probe sequences in Appendix Table 2). PCR testing was run on the Applied Biosystem 7500 Fast system. PCR master mix was made using the Quanta qScript XLT One-Step RT-qPCR ToughMix (Quantabio).

For viral sequencing, extracted RNA was converted to double-stranded cDNA, purified by bead cleanup, enzymatically fragmented, end-repaired, indexed, amplified, and purified again

using the QIAseq FX DNA Library Kit (Qiagen). Hybridization capture was performed using the QIAseq xHYB Viral Respiratory Panel (Qiagen) after pooling libraries by sample Crt values, with up to 6 samples in each pool. After overnight hybridization with biotinylated probes and subsequent washing to remove unbound fragments, enriched libraries were amplified and then purified by bead clean-up. Library fragment sizes were estimated by TapeStation 4200 D1000 (Agilent) and concentrations were measured by Qubit 4 Fluorometer (Invitrogen). Libraries passing quality control were sequenced on Illumina Novaseq 6000 or Nextseq 2000 instruments using a 2x150 read format. Consensus genomes were generated by using a custom bioinformatic pipeline (https://github.com/greninger-lab/revica) described previously (*2*). This pipeline performs trimming of raw reads for quality, reference selection, and iterative mapping to generate a consensus genome. Sequencing data has been uploaded to NCBI BioProject PRJNA1029161 (Accessions available in Appendix Table 1).

Statistical Analysis

We summarized demographic, clinical, and behavioral characteristics of households, index cases, and household contacts. The primary outcome was an **incident EV-D68 illness** defined as ≥1 respiratory symptom (e.g., fever, cough, sore throat, shortness of breath, myalgia, rhinorrhea)±7 days from a participant's first EV-D68-positive swab via multiplex PCR (**Day 0**). Symptom onset dates were not collected as part of the study symptom surveys; therefore, exact illness onset dates are not available for these analyses. Because the exact date of symptom onset could not be determined, we defined the start of illness using the first PCR–positive swab date. This approach was selected a priori to provide a consistent and objective definition of the primary case across households. An **individual illness episode** was defined as the period within which an individual's specimen(s) were PCR-positive for EV-D68 and the individual reported symptoms of cough, rhinorrhea, shortness of breath, nausea, headache, or fever with ≤14 days separating any 2 positive specimens. A **household illness episode** was defined as a period within which ≥1 individual illness episode(s) occurred in member(s) of the same household with ≤14 days separating EV-D68 positive specimens in the household. As household transmission can only be assessed when there are multiple household members present, single-person households were excluded from the analysis. Within households with any EV-D68 infection, the **index case** was defined as the first household member with EV-D68 detected. **Co-primary index cases** were defined as two or more household members with the same date of EV-D68 detection. A

household contact was defined as participants in the same household as an index case with a specimen(s) collected 1–14 days after the index case. The 14-day cutoff was based on other studies that have estimated the duration of EV-D68 shedding in the respiratory tract as 12 days (range 7 – 15 days) (*3*), and our prior work in other viruses like RSV (*4*). **Potential secondary transmission** was defined as additional household contact(s) with EV-D68 detected 1–14 days after the date of detection of the index case(s). **Unlikely secondary transmission** was defined as households with only one EV-D68 case detected, or households where a secondary case was detected >14 days after the index case, with no additional symptomatic EV-D68 detections in the intervening period. "The **symptomatic secondary infection rate** was defined as the probability that an infection occurs among susceptible people within the same household (*5*). We defined a **repeat detection** within the same individual as two detections of EV-D68 separated by >14 days with no positive results in the intervening period. **Detection interval** was defined as the number of days between the first positive specimen collection in the index case and the secondary case's first EV-D68 positive specimen collection. As participants swabbed weekly, we are unable to estimate serial intervals (days between index and secondary case symptom onset).

The primary outcome was secondary EV-D68 infection among household contacts within 14 days of an index case. The model predicting the symptomatic secondary infection rate and 95% confidence intervals used an intercept-only generalized estimating equation (GEE) to account for household clustering, and included household contacts of a distinct index case. All analyses were conducted in R version 4.3.2

The household income cutoff of $100,000 was determined by median household income in the Seattle and Portland metropolitan areas. The median household income in 2022 was $134,600 for Seattle and $106,000 for Portland. Based on city-specific statistics and the income survey question structure, the decision was made to make a binary cutoff of $100,000 for models included in this analysis.

Sequence Analysis

Trees were constructed using the Nextstrain platform (*6*) and made available via Nextstrain's community builds page (https://nextstrain.org/community/uwvirology-ngs/EV-D68/HouseholdTransmission) and Github (https://github.com/uwvirology-ngs/EV-D68). Consensus sequences were aligned using augur after filtering and subsampling to include all

study sequences and a random subset (5 per country-year-month) of contextual sequences downloaded from Genbank. Visualization was performed using auspice, and exported for annotation in ggtree (*7*). Pairwise comparison of sequences within the household was performed by aligning sequences using MAFFT (*8*), masking sites with ambiguities, and counting the number of pairwise nucleotide differences across the genome using the ape package (*9*) in R version 4.4.2.

References

1. Babu TM, Feldstein LR, Saydah S, Acker Z, Boisvert CL, Briggs-Hagen M, et al. CASCADIA: a prospective community-based study protocol for assessing SARS-CoV-2 vaccine effectiveness in children and adults using a remote nasal swab collection and web-based survey design. BMJ Open. 2023;13:e071446. PubMed https://doi.org/10.1136/bmjopen-2022-071446

2. Cox SN, Casto AM, Franko NM, Chow EJ, Han PD, Gamboa L, et al. Clinical and genomic epidemiology of coxsackievirus A21 and enterovirus D68 in homeless shelters, King County, Washington, USA, 2019–2021. Emerg Infect Dis. 2024;30:2250–60. PubMed https://doi.org/10.3201/eid3011.240687

3. Nguyen-Tran H, Thompson C, Butler M, Miller KR, Pyle L, Jung S, et al. Duration of enterovirus D68 RNA shedding in the upper respiratory tract and transmission among household contacts, Colorado, USA. Emerg Infect Dis. 2023;29:2315–24. PubMed https://doi.org/10.3201/eid2911.230947

4. Cox SN, Roychoudhury P, Frivold C, Acker Z, Babu TM, Boisvert CL, et al. Household transmission and genomic diversity of respiratory syncytial virus in the United States, 2022–2023. Clin Infect Dis. 2025;81:1159–69. https://doi.org/10.1093/cid/ciaf048

5. Liu Y, Eggo RM, Kucharski AJ. Secondary attack rate and superspreading events for SARS-CoV-2. Lancet. 2020;395:e47.

6. Hadfield J, Megill C, Bell SM, Huddleston J, Potter B, et al. Nextstrain: real-time tracking of pathogen evolution. Bioinformatics. 2018;34:4121–23.

7. Xu S, Li L, Luo X, Chen M, Tang W, Zhan L, et al. Ggtree: a serialized data object for visualization of a phylogenetic tree and annotation data. iMeta. 2022;1:e56. https://doi.org/10.1002/imt2.56

8. Katoh K, Standley DM. MAFFT multiple sequence alignment software version 7: improvements in performance and usability. Mol Biol Evol. 2013;30:772–80.

9. Paradis E, Claude J, Strimmer K. APE: Analyses of Phylogenetics and Evolution in R language. Bioinformatics. 2004;20:289–90. PubMed https://doi.org/10.1093/bioinformatics/btg412

Appendix Table 1. Sequenced samples

Household	State	Collection date	Age at swab, y	Accession no.
H1	WA	11/13/22	46	PV448643
H2	WA	9/18/22	39	PV448634
H3	WA	9/18/22	14	PV448642
H3	WA	9/25/22	48	PV448638
H4	WA	9/21/22	1	PV448650
H5	WA	9/10/22	43	PV448647
H6	WA	10/2/22	34	PV448639
H7	OR	8/21/22	35	PV648971
H8	OR	9/4/22	5	PV648965
H9	WA	7/24/22	4	PV448644
H10	WA	9/26/22	37	PV448640
H11	WA	9/11/22	4	PV521983
H12	OR	9/19/22	13	PV660520
H12	OR	9/26/22	46	PV648964
H13	OR	10/6/22	41	PV660514
H14	WA	9/23/22	6	PV448646
H15	WA	9/25/22	8	PV448637
H16	WA	9/12/22	1	PV448648
H17	WA	9/12/22	11	PX048941
H18	OR	9/18/22	36	PV660513
H18	OR	9/18/22	35	PV648972
H19	OR	9/4/22	49	PV648966
H20	OR	9/18/22	6	PV660519
H20	OR	9/18/22	42	PV648957
H21	OR	9/25/22	34	PV660517
H22	OR	9/7/22	37	PV660515
H23	OR	9/20/22	41	PV648961
H24	OR	9/18/22	13	PV660516
H25	WA	10/15/22	38	PX048942
H25	WA	10/15/22	0	PV448636
H26	WA	10/16/22	40	PV448645
H27	OR	10/10/22	10	PV660521
H28	OR	9/11/22	42	PV648967
H28	OR	9/11/22	41	PV648963
H29	OR	9/4/22	9	PV648958
H30	OR	10/3/22	33	PV648956
H31	OR	9/5/22	4	PV660511
H31	OR	9/5/22	35	PV648969
H32	OR	10/12/22	3	PV660523
H33	OR	9/11/22	11	PV660512
H34	OR	9/27/22	41	PV660522
H35	OR	9/28/22	36	PV648973
H36	OR	10/4/22	40	PV648962
H37	OR	9/18/22	37	PV648959
H38	OR	9/28/22	8	PV648970
H38	OR	9/28/22	37	PV648960
H39	OR	10/13/22	43	PX048940
H39	OR	10/13/22	42	PV660518
H39	OR	10/7/22	5	PV648968
H40	WA	10/25/22	2	PV448635
H41	WA	10/16/22	42	PV448641
H42	WA	10/27/22	37	PV448649

Appendix Table 2. EV-D68 PCR primers and probe sequences

Primer/Probe	Sequence
AN993	5′ GGAATAAATCCAGCNGAYACNAT 3′
AN995	5′ CCACGCTTTTATRTGYTTNGGYTTCAT 3′
AN992	5′ FAM GARCAYCARCCARTTGGTTTCACAGTGAC BHQ1 3′

Appendix Table 3. Demographic characteristics of households with and without a secondary EV-D68 infection within 14 d of a distinct index case during the incident household illness episode and where all household members were enrolled in the study, Washington and Oregon, United States, 2022–2024 (n = 15)*

Characteristic	Potential secondary transmission†	Unlikely secondary transmission‡	Total
Characteristics of household	(n = 2)	(n = 13)	(N = 15)
Household density§			
2–4 individuals	2 (100%)	13 (100%)	15 (100%)
Housing type			
House, condo, or townhouse	2 (100%)	13 (100%)	15 (100%)
Household enrollment			
Number of participants, median [min, max]	4 (2,4)	4 (2,4)	4 (2,4)
No child <5	0 (0%)	6 (46.2%)	6 (40.0%)
Child <5, but no childcare	1 (50.0%)	2 (15.4%)	3 (20.0%)
Child <5 in childcare¶	1 (50.0%)	5 (38.5%)	6 (40.0%)
Income $100,000+	1 (50.0%)	10 (76.9%)	11 (73.3%)
Smoker in household	1 (50.0%)	1 (7.7%)	2 (13.3%)
Study site			
Kaiser Permanente Northwest (KPNW)	1 (50.0%)	5 (38.5%)	6 (40.0%)
University of Washington (UW)	1 (50.0%)	8 (61.5%)	9 (60.0%)
Characteristics of index case	(n = 2)	(n = 13)	(N = 15)
Age			
Median [min, max] years	18.5 [3, 34]	4 [1, 48]	4 [1, 48]
6 mo–1 y	0 (0.0%)	1 (7.7%)	1 (6.7%)
2–4 y	1 (50.0%)	6 (46.2%)	7 (46.7%)
5–12 y	0 (0%)	4 (30.8%)	4 (26.7%)
13–50 y	1 (50.0%)	2 (15.4%)	3 (20.0%)
Female sex at birth	1 (50.0%)	8 (61.5%)	9 (60.0%)
Gender			
Female	1 (50.0%)	8 (61.5%)	9 (60.0%)
Male	1 (50.0%)	5 (38.5%)	6 (40.0%)
Race			
White	1 (50.0%)	12 (92.3%)	13 (86.7%)
Multiracial	1 (50.0%)	1 (7.7%)	2 (13.3%)
Any comorbidities#	1 (50.0%)	3 (23.1%)	4 (26.7%)
Masking in public			
Any	2 (100%)	11 (84.6%)	13 (86.7%)
Never	0 (0.0%)	2 (15.4%)	2 (13.3%)
Relative cycle threshold (C_{rt})			
Median [Min, Max]	17.6 [15.5, 19.7]	15.3 [9.6, 22.3]	15.5 [9.6, 22.3]
≤median (19.9 C_{rt})	2 (100%)	10 (76.9%)	12 (80.0%)
Presence of viral codetection			
Any	2 (100%)	11 (84.6%)	13 (86.7%)
Rhinovirus	2 (100%)	11 (84.6%)	13 (86.7%)
Adenovirus	1 (50.0%)	0 (0.0%)	1 (6.7%)
HPIV	1 (50.0%)	0 (0.0%)	1 (6.7%)
ARI symptoms			
2+ ARI symptom(s)**	2 (100%)	10 (76.9%)	12 (80.0%)
Cough and/or rhinorrhea	2 (100%)	13 (100%)	15 (100%)
Any care seeking during illness††	0 (0.0%)	2 (15.4%)	2 (13.3%)
Any behavior change to reduce transmission in the air (e.g., masking)‡‡	1 (50.0%)	4 (30.8%)	5 (33.3%)
Any behavior change to reduce transmission on surfaces§§	1 (50.0%)	2 (15.4%)	3 (20.0%)
Characteristics of household contacts	(n = 6)	(n = 30)	(n = 36)
Age			
Median [min, max] years	23 [2, 42]	39 [5, 47]	38.5 [2, 47]

Characteristic	Potential secondary transmission†	Unlikely secondary transmission‡	Total
2–4 y	1 (16.7%)	0 (0.0%)	1 (2.8%)
5–12 y	2 (33.3%)	8 (26.7%)	10 (27.8%)
13–50 y	3 (50.0%)	22 (43.3%)	25 (69.5%)
Female sex at birth	3 (50.0%)	16 (53.3%)	19 (52.8%)
Gender			
Female	3 (50.0%)	15 (50.0%)	18 (50.0%)
Male	3 (50.0%)	14 (46.7%)	17 (47.2%)
Other	0 (0.0%)	1 (3.3%)	1 (2.8%)
Race			
Asian	1 (16.7%)	1 (3.3%)	2 (5.6%)
White	4 (66.7%)	28 (93.3%)	32 (88.9%)
Multiracial	1 (16.7%)	1 (3.3%)	2 (5.6%)
Any smoking	1 (16.7%)	1 (3.3%)	2 (5.6%)
Any comorbidities#	3 (50.0%)	13 (43.3%)	16 (44.4%)
Masking in public			
Any	6 (100%)	27 (90.0%)	33 (91.7%)
Never	0 (0.0%)	3 (10.0%)	3 (8.3%)

*ARI, acute respiratory illness; Crt, relative cycle threshold; EV, enterovirus; HPIV, human parainfluenza virus; RSV, respiratory syncytial virus.
†Potential secondary transmission defined as occurring when a secondary household member with EV-D68 is detected 1-14 days after index case.
‡Unlikely secondary transmission defined as occurring when there is only 1 EV-D68 case detected in the household OR when there are 2+ EV-D68 cases in a household but secondary case detected >14 days after index case.
§Household density represents the number of household members regardless of enrollment in the study (thus, this may be greater than the number of individuals enrolled in the study if not all household members are enrolled).
¶Daycare or school attendance among at least one child <5 years in the household as reported at enrollment
#Comorbidities include asthma, chronic obstructive pulmonary disease (COPD) (including chronic bronchitis and emphysema), sleep apnea, heart disease, congenital heart disease, heart failure, down syndrome, hypertension (high blood pressure), diabetes (high blood sugar), liver condition, weak or failing kidneys, cancer or malignancy, arthritis, stroke, deep vein thrombosis (DVT) or pulmonary embolism (PE), sickle cell disease or thalassemia, weakened immune system, depression, anxiety, thyroid issues, or other health diagnosis.
**ARI symptom reported within ±7 days of the individual EV-D68 illness episode's first positive specimen collection, including fever, cough, sore throat, shortness of breath, myalgia, and/or rhinorrhea.
††Self-reported care seeking defined as seeking health care (from a healthcare provider) during illness
‡‡Includes masking, sleeping separately, covering cough/sneeze
§§Includes handwashing, cleaning, disinfecting

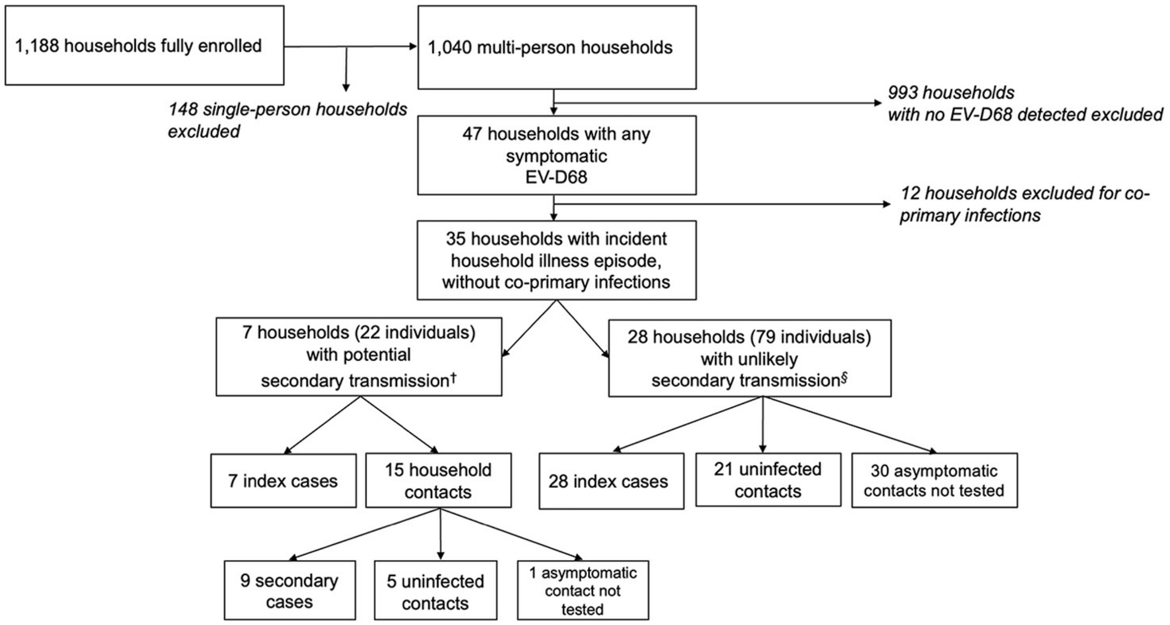

Appendix Figure 1. Study Flow Diagram. †Potential secondary transmission = household member with EV-D68 is detected 1–14 d after index case. §Unlikely secondary transmission only 1 EV-D68 case detected in the household or ≥2 EV-D68 cases in a household but secondary case detected >14 d after index case.

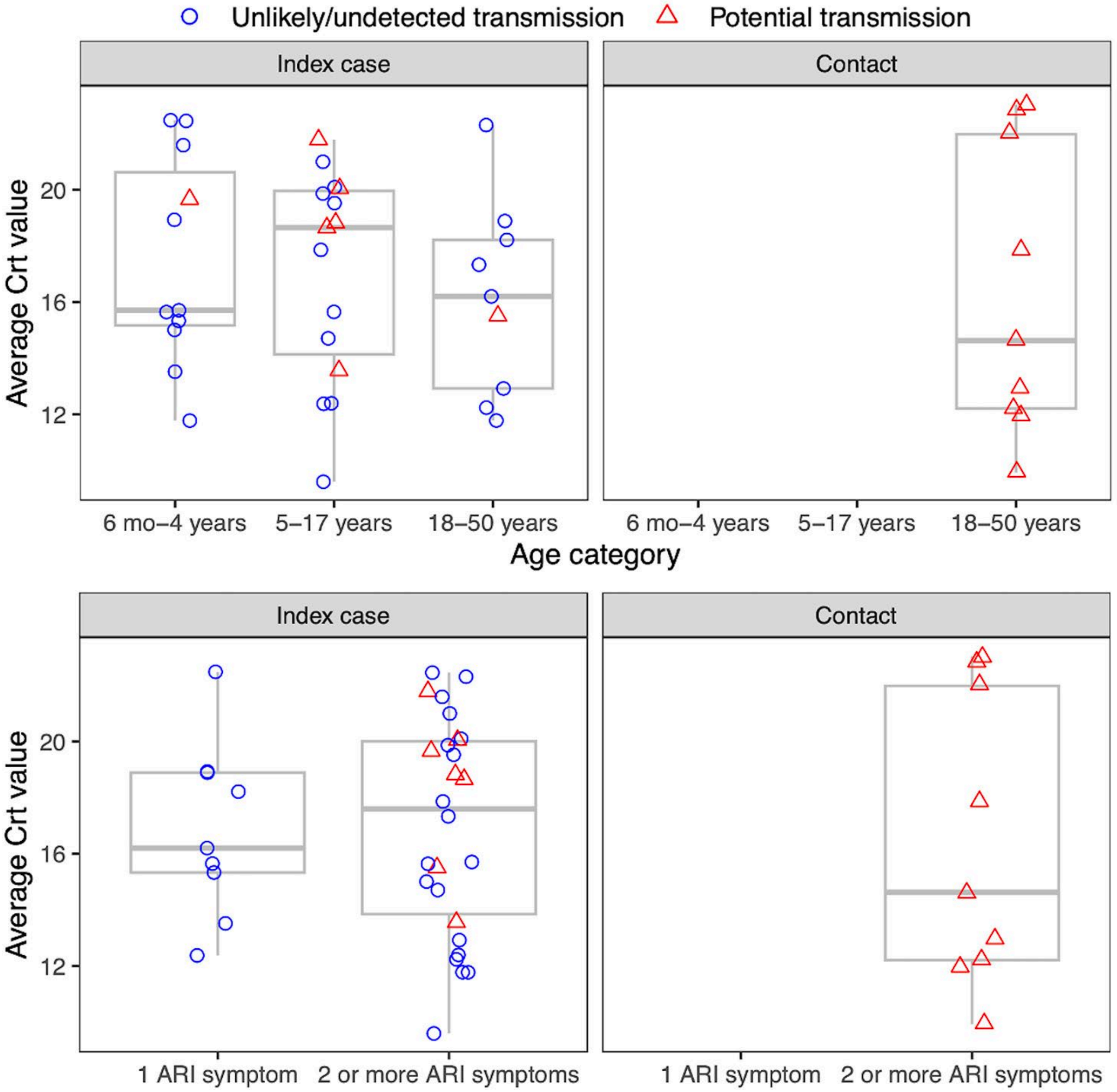

Appendix Figure 2. Comparing Crt values across age groups (top panel) and by number of reported ARI symptoms (bottom panel) split by index cases and household contacts.

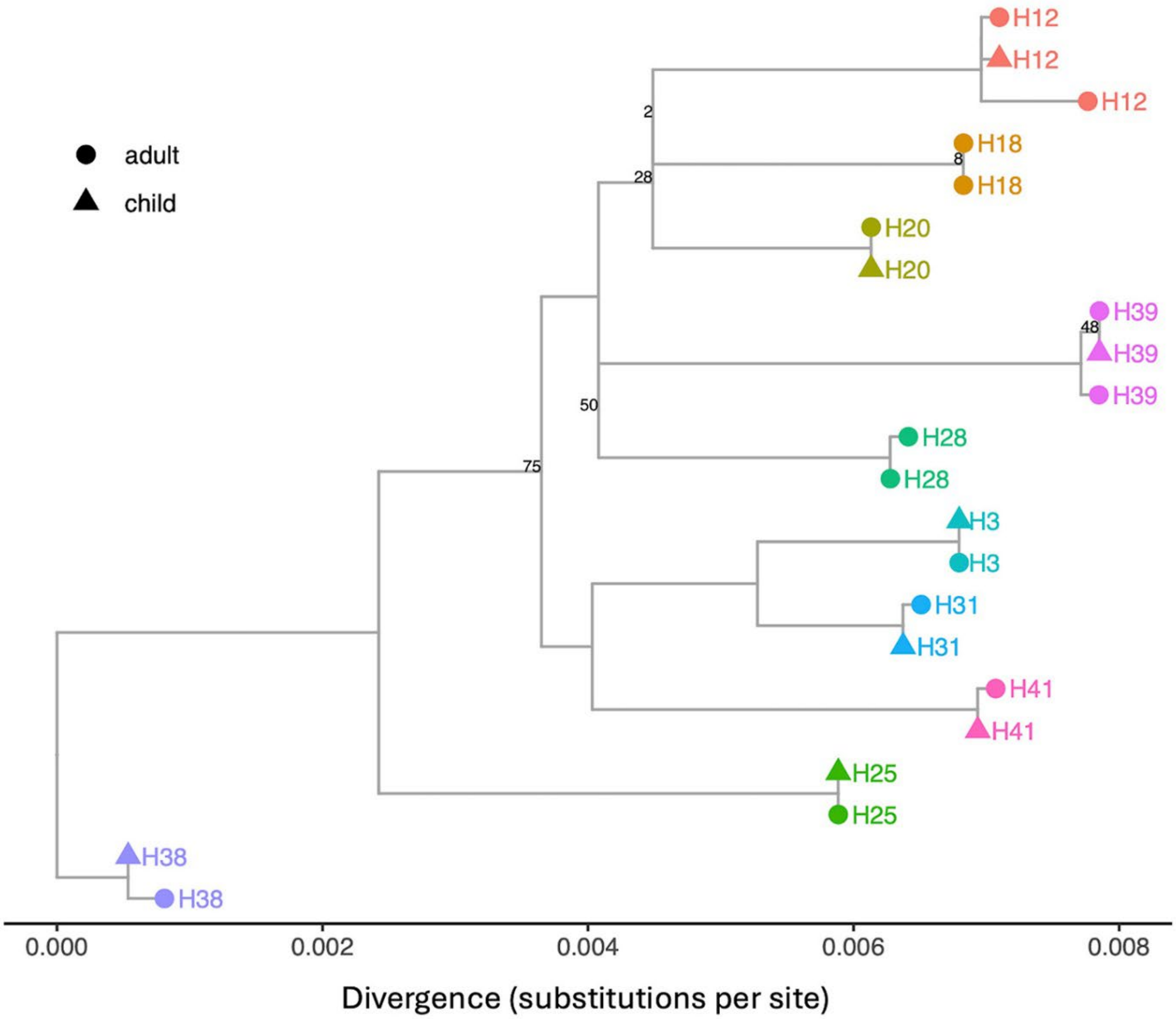

Appendix Figure 3. Maximum likelihood tree for sequences from 10 households with samples collected 0–14 days apart. Tip color indicates household, tip shape indicates whether the sample was from an adult or child. Bootstrap values shown only for nodes with <80% support.

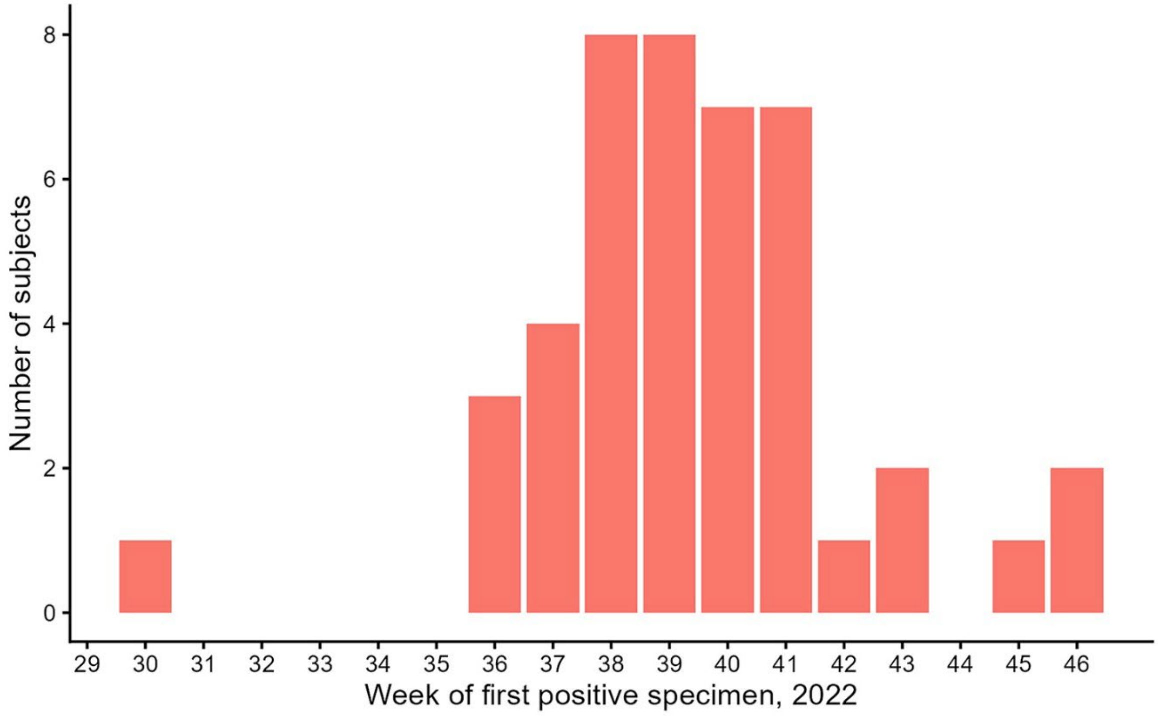

Appendix Figure 4. Count of incident EV-D68 cases, by CDC epidemiologic week in 2022.
